# Discovery of structural and functional transition sites for membrane-penetrating activity of sheep myeloid antimicrobial peptide-18

**DOI:** 10.1038/s41598-023-28386-6

**Published:** 2023-01-23

**Authors:** Bomi Jung, Hyosuk Yun, Hye Jung Min, Sungtae Yang, Song Yub Shin, Chul Won Lee

**Affiliations:** 1 Department of Chemistry, Chonnam National University, Gwangju, 61186 Republic of Korea; 2 Department of Cosmetic Science, Gwangju Women’s University, Gwangju, 62396 Republic of Korea; 3 Department of Microbiology, School of Medicine, Chosun University, Gwangju, 61452 Republic of Korea; 4 Department of Cellular and Molecular Medicine, School of Medicine, Chosun University, Gwangju, 61452 Republic of Korea

**Keywords:** Peptides, Infectious diseases, NMR spectroscopy

## Abstract

Cathelicidin antimicrobial peptides have an extended and/or unstructured conformation in aqueous solutions but fold into ordered conformations, such as the α-helical structure, when interacting with cellular membranes. These structural transitions can be directly correlated to their antimicrobial activity and its underlying mechanisms. SMAP-18, the N-terminal segment (residues 1–18) of sheep cathelicidin (SMAP-29), is known to kill microorganisms by translocating across membranes and interacting with their nucleic acids. The amino acid sequence of SMAP-18 contains three Gly residues (at positions 2, 7, and 13) that significantly affect the flexibility of its peptide structure. This study investigated the role of Gly residues in the structure, membrane interaction, membrane translocation, and antimicrobial mechanisms of SMAP-18. Five analogs were designed and synthesized through Gly → Ala substitution (i.e., G2A, G7A, G13A, G7,13A, and G2,7,13A); these substitutions altered the helical content of SMAP-18 peptides. We found that G7,13A and G2,7,13A changed their mode of action, with circular dichroism and nuclear magnetic resonance studies revealing that these analogs changed the structure of SMAP-18 from a random coil to an α-helical structure. The results of this experiment suggest that the Gly residues at positions 7 and 13 in SMAP-18 are the structural and functional determinants that control its three-dimensional structure, strain-specific activity, and antimicrobial mechanism of action. These results provide valuable information for the design of novel peptide-based antibiotics.

## Introduction

The emergence of multidrug-resistant bacteria has created a need to develop novel classes of antibiotics to circumvent them^1^. As part of these efforts, antimicrobial peptides (AMPs) are considered a potential alternative to existing antibiotics^2,3^. AMPs are active against a wide range of pathogenic microorganisms^4,5^ and have already shown clinical success^6–8^. AMPs can disrupt bacterial membranes by forming toroidal pores^4^; some polar or charged residues on the hydrophobic face of AMPs can promote the formation of pores by drawing lipid-head groups into the bacterial membrane. Pore formation is often connected to a local change in membrane thickness^9^. Under the carpet model, peptides are accumulated on the bacterial membrane surface^10^, with AMPs working similarly to detergents and breaking off membrane lipids into micelle-like structures; this model is commonly considered an extreme extension of the toroidal pore model. Within the barrel-stave model, peptide helices form a barrel-shaped bundle in the membrane composed of helical peptides as the staves^11,12^. The hydrophobic peptide regions align with the core of the lipid bilayer and the hydrophilic peptide regions form the internal regions of the pore^12,13^. Although antimicrobial mechanisms mainly involve disturbing the cytoplasmic membrane in various manners, other modes of action that target key intracellular processes such as DNA and protein synthesis, protein folding, enzymatic activity and cell wall synthesis have recently been identified^4,14,15^. For example, unlike most α-helical peptides, buforin II does not induce membrane disruption^16^; it accumulates in the cytoplasm where it exert antimicrobial activity by adopting an extended or polyproline II conformation when bound to DNA^17^.

AMP structures are related to antimicrobial activity and selective toxicity. The structure of AMP is determined by the following structural parameters: size, cationicity, structure, hydrophobicity, hydrophobic moment, amphipathicity, and hydrophobic or polar angle^18^.

These molecular determinants are relative to one another, i.e., modifying one parameter often results in compensatory changes to other parameters. A holistic view of peptide structure–activity relationships is associated with each of these key properties that affect the mode of action of AMPs^19^. Amphipathic faces of α-helical AMPs are separated along the α-helix axis, making the peptides parallel to the membrane plane during initial lipid interactions, the charged surface facing the phospholipid head groups, and the hydrophobic surface embedded in the acyl tail core^3^. The properties of this α-helical structure occasionally affects membrane insertion, thus affecting the antimicrobial activity^20^.

**Figure 1:**
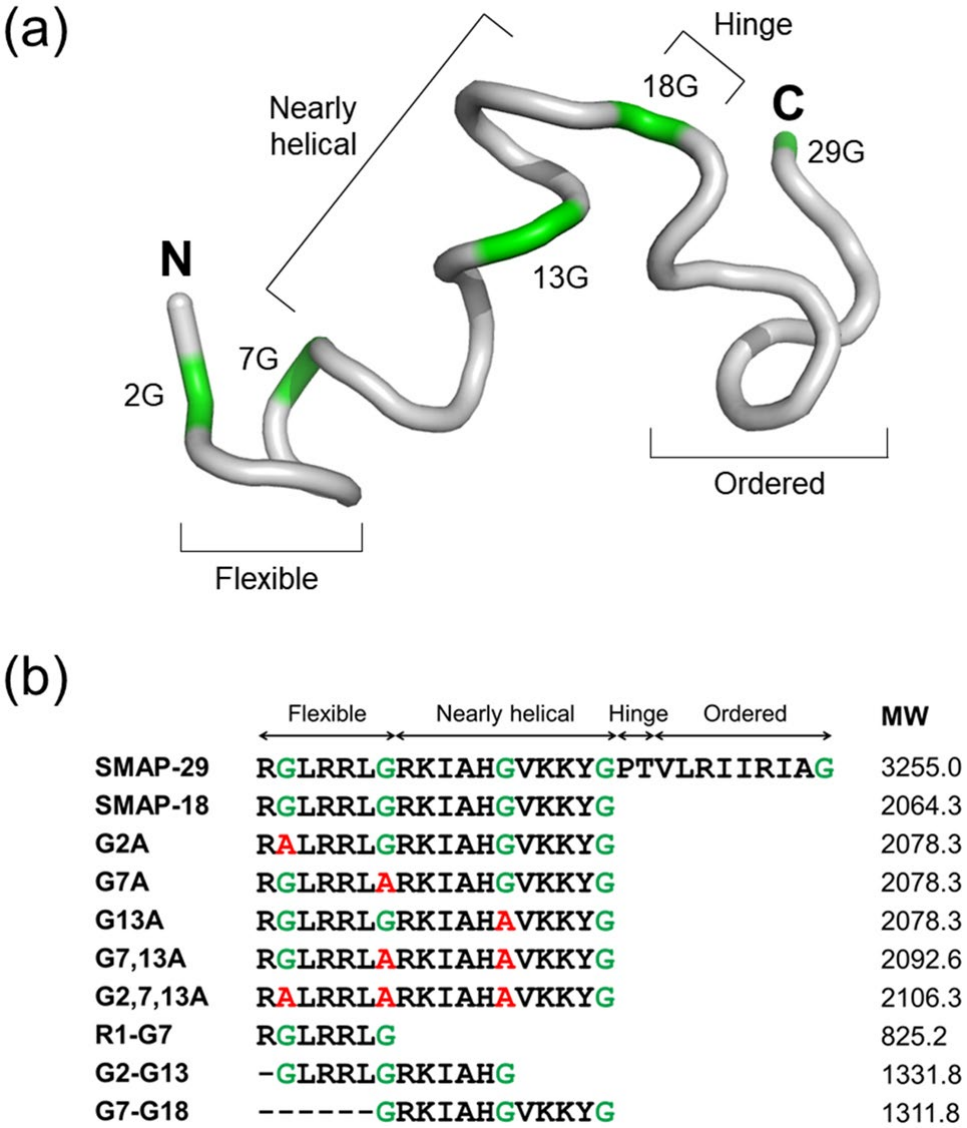
Three-dimensional structure of SMAP-29 and amino acid sequences of SMAP-18 analogs. (**a**) NMR solution structure of SMAP-29 solved in 40% trifluoroethanol (PDB ID 1FRY). The N-terminal flexible, nearly helical, hinge, and C-terminal ordered regions are indicated. The glycine residues are colored in green. (**b**) The amino acid sequences and molecular weights of SMAP-18 analogs synthesized in this study.

Sheep myeloid antimicrobial peptide-29 (SMAP-29) is a member of cathelicidin family of AMPs, displaying potent broad-spectrum antimicrobial activity against gram-negative and gram-positive bacteria and fungi^21^. SMAP-29 is known to kill microorganisms by disrupting/perturbing their lipid bilayer and by forming pores/ion channels on bacterial cell membranes^22^. In contrast, SMAP-29 is very cytotoxic to mammalian cells such as human red blood cells (hRBCs) and human embryonic kidney (HEK) cells. The high hemolytic activity of SMAP-29 is due to its hydrophobic C-terminal region (residues 19–29). The N-terminal 18-mer peptide (i.e., SMAP-18) of SMAP-29 showed good selectivity against bacterial cells due to its dramatic reduction in hemolytic activity and antimicrobial retention activity^23^. Similar to buforin II, SMAP-18 was found to kill microorganism through intracellular-targeting mechanism^24^; SMAP-18 can penetrate across the bacterial cell membranes causing damage. The 3-D structure of SMAP-29 is divided into three segments– a highly flexible region from the N-terminus to a 'hinge' at Gly^7^, a helical region from Arg^8^ to Tyr^17^, and another nearly helical region from Thr^20^ to Ala^28^ following the 'hinge' at Gly^18^/Pro^19^^25^ (see Fig. 1a). In many cases, AMPs that retain antimicrobial activity but have low cytotoxicity require a membranous environment to induce proper folding and often have a Pro- or Gly-induced kink in the center of the α-helix^3^. In particular, Gly is known to be a more flexible residue than other amino acids and acts as a helix breaker. The relationships between the structural features of membrane-active peptides and the mechanism of their action are not clear.

SMAP-18 contains four Gly residues at positions 2, 7, 13, and 18. Gly^18^ at the C-terminus of SMAP-18 is not expected to have significant influence over structural features. Thus, this study investigated the effects three Gly residues (at positions 2, 7, and 13) on the tertiary structure of the peptide and the bacteria-killing mechanism of Gly → Ala substitution to better understand the relationship between structural flexibility and membrane interaction. We designed and synthesized a series of five Gly → Ala substituted analogs of SMAP-18 and compared the structure and antimicrobial activities of these SMAP-18 peptides. The mechanism of action of these peptides was also investigated in this study.

## Results

### Design and synthesis of SMAP-18 analogs

To assess the structural and functional roles of Gly residues in SMAP-18, we designed and synthesized various SMAP-18 analogs, including Gly → Ala substitutes and short truncated peptides (see Fig. 1b). SMAP-18 contains four Gly residues, but since the Gly^18^ at the C-terminus was not expected to have a significant structural and functional influence, it has been excluded from consideration during this research. This study focused on the Gly^2^, Gly^7^, and Gly^13^ residues. For the single substitution, G2A, G7A and G13A peptides were synthesized. G7,13A (i.e., double point mutation) and G2,7,13A (i.e., triple point mutation) were synthesized by Gly^7,13^ → Ala and Gly^2,7,13^ → Ala substitutions. To investigate the role of N- or C-terminal region divided by Gly in SMAP-18, short truncated peptides (i.e., R1-G7, G2-G13, and G7-G18) were designed and synthesized. The sequences and molecular weights of SMAP-18 peptides are summarized in Fig. 1b. All peptides were purified through preparative reversed phase-HPLC (RP-HPLC). The molecular weights and purities of the synthesized peptides were confirmed through liquid chromatography–mass spectrometry (LC–MS) analysis (see Fig. S1).

### Antimicrobial activity of SMAP-18 analogs

**Figure 2:**
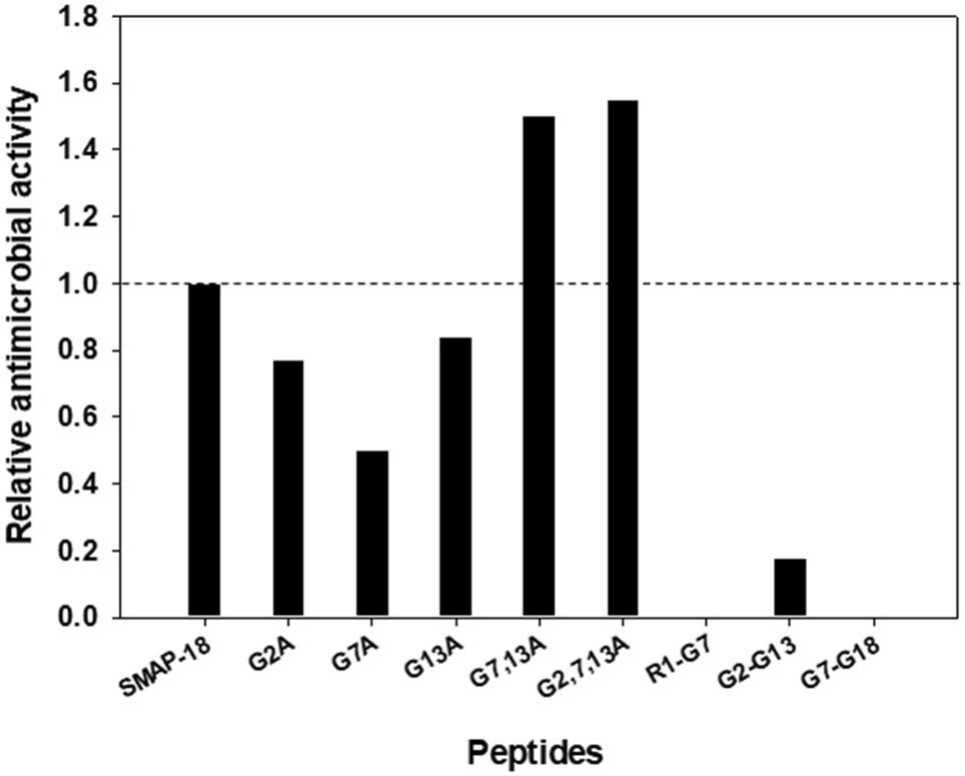
Relative antimicrobial activity of SMAP-18 analogs compared to wild-type SMAP-18 produced based on Table 1.

To test the antimicrobial activities of SMAP-18 and its analogs, we determined the minimum inhibitory concentration (MIC) value against three gram-negative bacteria (i.e., *Escherichia coli, Salmonella typhimurium* and *Pseudomonas aeruginosa*) and three gram-positive bacteria (i.e., *Bacillus subtilis, Staphylococcus epidermidis* and *Staphylococcus aureus*) (see Table 1). SMAP-18 showed high antimicrobial activity against both gram-positive and gram-negative bacteria displaying MIC values from 4 to 8 μM with a GM value of 6.7. The single site mutants (i.e., G2A, G7A, and G13A) showed similar or 2- to fourfold decreases in antimicrobial activity against both gram-negative and gram-positive bacteria compared to SMAP-18. The antimicrobial activity of G7,13A increased 2- to eightfold against all bacterial strains except *E. coli* and *S. epidermidis*. G2,7,13A exhibited 2- to fourfold higher antimicrobial activity against all bacteria except *S. aureus* and *S. epidermidis*. Based on GM values, the single site substitution of Gly to Ala decreased antimicrobial activity by up to twofold (see Fig. 2). In contrast, double or triple substituted site mutants slightly increased their antimicrobial activity, up to 1.5-fold. These results suggested that single site mutations may disrupt the active conformation of SMAP-18, but multiple site mutations may induce more active conformation for more effective antimicrobial activity of SMAP-18. Furthermore, N- or C-terminal truncated peptides significantly decreased their antimicrobial activity, suggesting that the length of short truncated peptides is not enough to exhibit effective antimicrobial activity.

**Table 1:** MIC values (μM) of SMAP-18 and SMAP-18 analogues against Gram-negative and Gram-positive bacteria.

	SMAP-18	G2A	G7A	G13A	G7,13A	G2,7,13A	R1-G7	G2-G13	G7-G18
Gram-negative									
* E. coli*	4	8	16	4	8	2	> 64	32	> 64
* S. typhimurium*	8	8	16	8	4	4	> 64	32	> 64
* P. aeruginosa*	8	8	16	8	4	2	> 64	32	> 64
Gram-positive									
* B. subtilis*	8	8	16	16	1	2	> 64	32	> 64
* S. epidermidis*	4	4	8	4	8	8	> 64	32	> 64
* S. aureus*	8	16	8	8	2	8	> 64	64	> 64
GM^a^	6.7(± 2.1)	8.7(± 3.9)	13.3(± 4.1)	8.0(± 4.4)	4.5(± 2.9)	4.3(± 2.9)	> 64	37.3(± 13.1)	> 64

^a^GM denotes the geometric mean of MIC values from selected bacterial strains (average value ± SD).

### CD spectra

**Figure 3:**
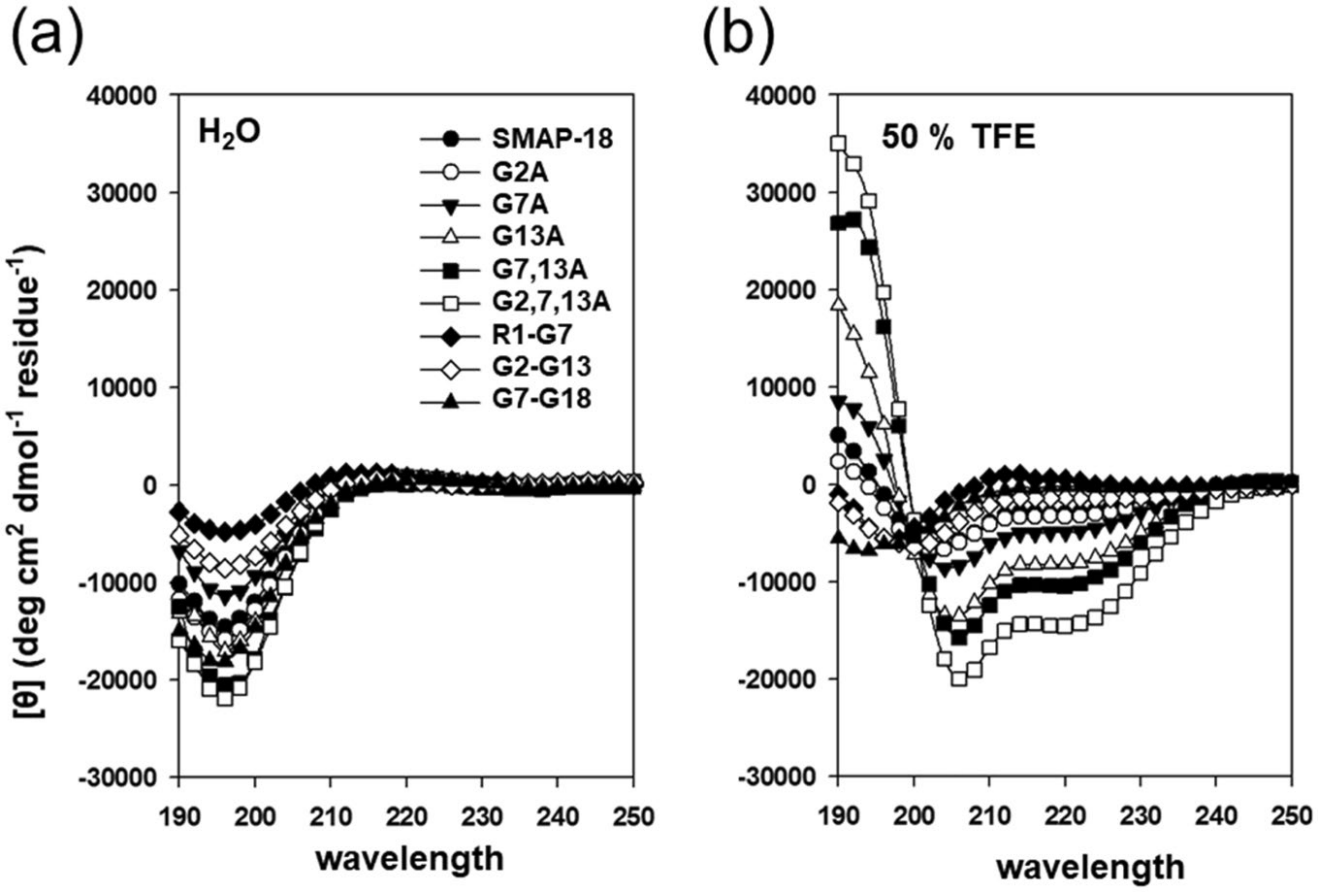
CD spectra of SMAP-18 analogs in H_2_O (10 mM sodium phosphate, pH 7.0) (**a**) and 50% TFE (**b**). The peptide concentration is 100 μM. Three times scans were accumulated from 190 to 250 nm.

Figure 3 shows the circular dichroism (CD) spectra of SMAP-18 and SMAP-18 analogs in H_2_O and 50% trifluoroethanol (TFE). In H_2_O (10 mM sodium phosphate, pH 7.0), the spectra of all peptides indicated that those peptides exhibited random coil conformation. In the 50% TFE cell membrane-like environment, the spectra of SMAP-18 and analogs that contained Ala substitutions for Gly showed negative maxima at around 208 nm and 220 nm, indicating the significance of the α-helix structure; those peptides exhibited conformational changes. The estimation of helicity from the CD spectra is summarized in Table 2. There were no significant structural differences between SMAP-18 and G2A, but the substitution of Ala for Gly^7^ and Gly^13^ of SMAP-18 resulted in an increase in the α-helical structure. When a single Gly molecule was substituted with Ala, G13A had higher α-helicity than G7A. In addition, the more Gly that is replaced with Ala, the greater is the tendency to increase α-helical formation. The spectrum of the truncated analogs did not show an α-helical structure, even in 50% TFE.

**Table 2:** Estimation of helicity from CD of SMAP-18 and SMAP-18 analogs.

Peptides	% α-helix	
	H_2_O	50% TFE
SMAP-18	6.6	15.3
G2A	6.6	16.1
G7A	5.9	20.0
G13A	7.0	28.4
G7,13A	7.6	33.9
G2,7,13A	5.7	44.4
R1-G7	5.6	6.9
G2-G13	6.6	11.5
G7-G18	6.6	8.3

### NMR analysis

**Figure 4:**
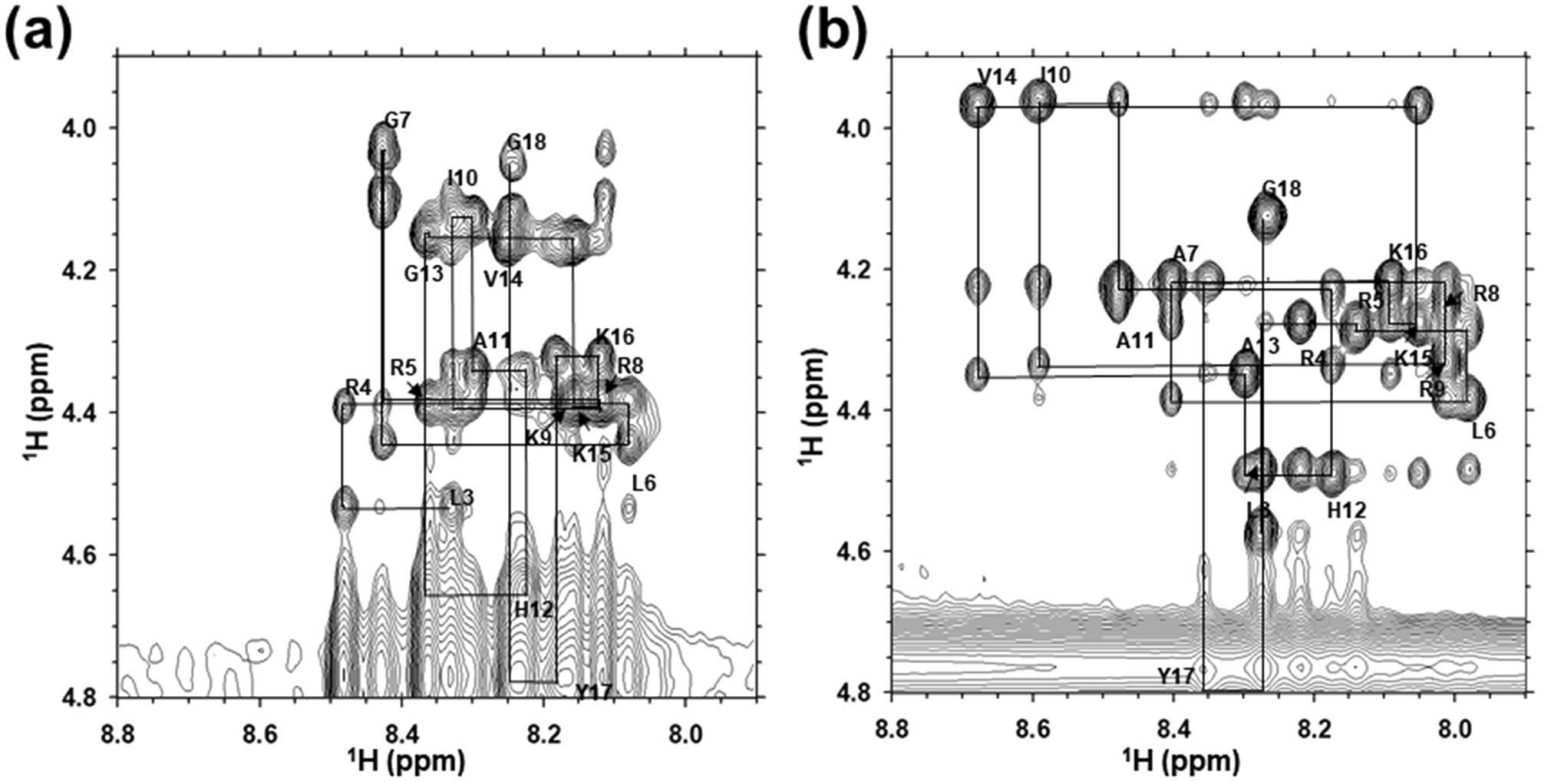
Sequential d_αN_(*i, i* + *1*) NOE connectivity for residues from 3 to 18 of SMAP-18 (**a**) and G2,7,13A analog (**b**).

Nuclear magnetic resonance (NMR) experiments were performed in a 50% TFE environment. TFE can induce the formation of stable α-helical conformations of peptides that are otherwise unstructured in a 100% aqueous solution^26^. Complete proton resonance assignments for SMAP-18 and G2,7,13A were determined using 2D NMR spectroscopy. Chemical shift assignments of SMAP-18 and G2,7,13A were based on scalar coupling patterns observed in double quantum-filtered correlation spectroscopy (DQF-COSY) and total correlation spectroscopy (TOCSY) spectra, which complemented the results of nuclear overhauser effect spectroscopy (NOESY) measurements. Table 3 shows the NMR proton chemical shifts of SMAP-18 and G2,7,13A. The identified spin systems were aligned along the primary structure of SMAP-18 and G2,7,13A through inter-residue sequential NOEs observed on the NOESY spectrum. Figure 4 shows the NOESY spectra with sequential d_αN_(*i, i* + 1) connectivity. The pattern of observed NOEs was analyzed in terms of the secondary structure of the peptides. As summarized in Fig. S2 of the supplementary material, medium-distance NOEs were observed in both SMAP-18 and G2,7,13A. G2,7,13A in particular, showed typical α-helical NOE patterns similar to those of d_αN_(*i, i* + 3), d_αβ_(*i, i* + 3), d_αN_(*i, i* + 4), indicating that the α-helix structure is comprised of residues 3–17.

**Table 3:** NMR proton chemical shifts of SMAP-18 and G2,7,13A.

Sequence	Residue	NH	Hα	Hβ	Other
SMAP-18					
1	Arg	ND	ND	ND	
2	Gly	ND	ND	ND	
3	Leu	8.3267	4.5292	1.8539	γCH 1.8539; δCH_3_ 1.1299, 1.0846
4	Arg	8.4776	4.3904	2.1118	γCH_2_ 1.9600, 1.8524; δCH_2_ 3.4089
5	Arg	8.3599	4.3807	2.0852	γCH_2_ 1.9249, 1.8457; δCH_2_ 3.4119
6	Leu	8.0792	4.4388	1.9173, 1.8670	γCH 1.8670; δCH_3_ 1.1429, 1.0888
7	Gly	8.4250	4.0920, 4.0332		
8	Arg	8.1133	4.3804	2.0994	γCH_2_ 1.9569, 1.8501; δCH_2_ 3.3980
9	Lys	8.1625	4.3883	2.1660	γCH_2_ 1.7685, 1.6499; δCH_2_ 1.8981; ɛCH_2_ 3.1754
10	Ile	8.3180	4.1229	2.1000	γCH_2_ 1.3649, 1.8265; γCH_3_ 1.0853; δCH_3_ 1.0362
11	Ala	8.2929	4.3389	1.6207	
12	His	8.2215	4.6441	3.4176, 3.4466	
13	Gly	8.3637	4.1449		
14	Val	8.2432	4.1576	2.3474	γCH_3_ 1.1413, 1.2003
15	Lys	8.1562	4.3799	2.0067	γCH_2_ 1.6568, 1.6069; δCH_2_ 1.8809; ɛCH_2_ 3.1594
16	Lys	8.1196	4.3206	1.8122	γCH_2_ 1.3977, 1.2886; δCH_2_ 1.7730; ɛCH_2_ 3.1122, 3.0965
17	Tyr	8.1777	4.7825	3.3468, 3.1284	2,6H 7.3452; 3,5H 7.0132
18	Gly	8.2414	4.1068, 4.0495		
G2,7,13A					
1	Arg	ND	ND	ND	
2	Ala	ND	4.5804	1.6751	
3	Leu	8.2807	4.4936	1.8934	γCH 1.8938; δCH3 1.1246, 1.1466
4	Arg	8.2270	4.2866	2.1474	γCH2 1.9639, 1.8808; δCH2 3.4418
5	Arg	8.1469	4.2953	2.1453	γCH2 2.0059, 1.8706; δCH2 3.4406 εNH 7.5415
6	Leu	7.9899	4.3671	2.0821, 1.8730	γCH 1.8723; δCH3 1.1611, 1.1121
7	Ala	8.4097	4.2270	1.6958	
8	Arg	8.0229	4.2656	2.1582	γCH2 2.0525; δCH2 3.4326
9	Lys	8.0214	4.3431	2.2841	γCH2 1.8670 1.6884; δCH2 1.9254; εCH2 3.1876
10	Ile	8.5944	3.9712	2.1256	γCH2 1.3352; γCH3 1.1565; δCH3 1.0219
11	Ala	8.4829	4.2324	1.6880	
12	His	8.1794	4.5016	3.5312	
13	Ala	8.3039	4.3543	1.8390	
14	Val	8.6785	3.9782	2.3813	γCH3 1.1446, 1.2581
15	Lys	8.0595	4.2901	2.0850	γCH2 1.7369 1.6480; δCH2 1.8856; εCH2 3.1723
16	Lys	8.0991	4.2270	1.7561	γCH2 1.3355 1.1221; δCH2 1.7132; εCH2 3.0856
17	Tyr	8.3530	4.8038	3.4200, 3.1259	2,6H 7.4047; 3,5H 7.0223
18	Gly	8.2736	4.1313

### NMR structure calculation of SMAP-18 and G2,7,13A

To calculate the 3-D structure of SMAP-18 and G2,7,13A, we used Cyana 2.1^27^ with automatic NOE assignment. Experimental constraints for SMAP-18 and G2,7,13A calculation included distance restraints, such as sequential and medium-range NOE restraints. We selected 20 structures based on the low values of target function. Structural statistics for these final 20 structures were evaluated using structural parameters and the low root mean square deviations (RMSD) were estimated (see Table 4). The atomic RMSD of the mean structure of SMAP-18 for residues 3–15 were 0.32 ± 0.17 Å for the backbone atoms (i.e., N, C^α^, and C) and 1.14 ± 0.23 Å for all heavy atoms. The atomic RMSD of the mean structure of G2,7,13A for residues 3–15 were 0.07 ± 0.03 Å for backbone atoms and 0.78 ± 0.16 Å for all heavy atoms.

**Table 4:** Structural statistics for the 20 lowest-target function structures of SMAP-18 and G2,7,13A.

	SMAP-18	G2,7,13A
Distance restraints		
Sequential	206	247
Medium-range	63	128
Other	6	22
Total	275	397
Mean cyana target function (Å^2^)	0.0347$\pm$0.0020	0.0047$\pm$0.0002
RMS deviation from mean structure (residues 1–18) (Å)		
Backbone atoms	1.22$\pm$0.39	0.44$\pm$0.13
Heavy atoms	2.48$\pm$0.56	1.45$\pm$0.29
RMS deviation from mean structure (residues 3–15) (Å)		
Backbone atoms	0.32$\pm$0.17	0.07$\pm$0.03
Heavy atoms	1.14$\pm$0.23	0.78$\pm$0.16
Ramachandran plot for the mean structure		
Residues in the most favorable allowed region (%)	72.8	83.4
Residues in the additionally allowed region (%)	26.6	16.6
Residues in the disallowed region (%)	0	0

### Structural description of SMAP-18 and G2,7,13A

**Figure 5:**
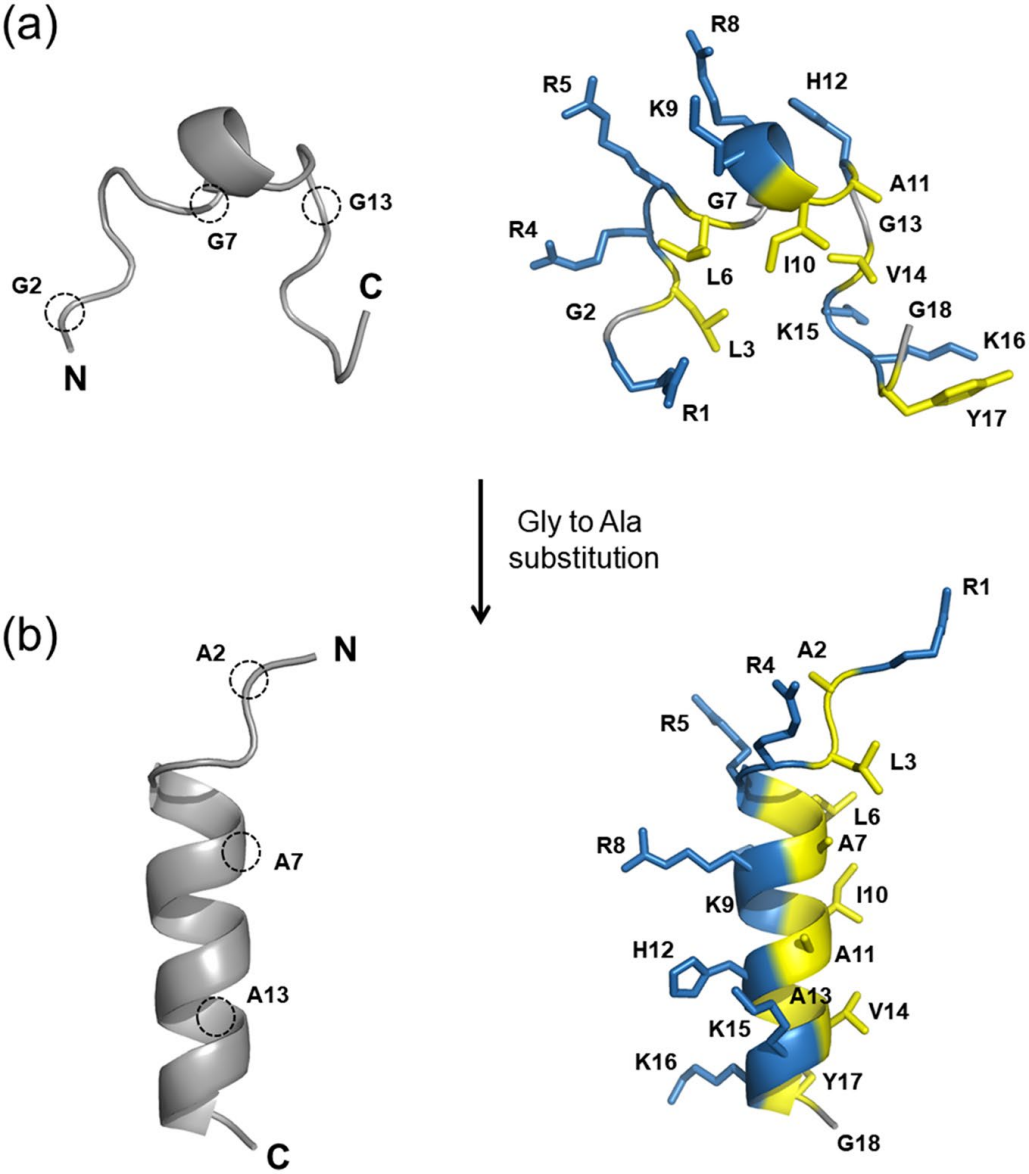
NMR solution structures of SMAP-18 (**a**) and G2,7,13A (**b**). Ribbon structures was shown with side chains and residue numbers. The glycine residues (G2, G7, and G13) are indicated by dotted circles. Hydrophobic and positively charged residues are colored in yellow and blue, respectively.

A short helical region (i.e., residues 6–10) is found in the center of the SMAP-18 structure but the length of this helix is too short to form a typical stable α-helical structure (see Fig. 5a). The helical content of SMAP-18 was only 15.3%, as calculated through CD spectrum analysis as well as 50% TFE (see Table 2). The backbone structure of SMAP-18 displayed predominantly unstructured-like loop characteristics (see Fig. 5a). In particular, Gly residues at positions 2, 7, and 13 are placed in the unstructured region of SMAP-18. In the case of G2,7,13A, wherein Gly was replaced with Ala, the aforementioned region is folded into a typical α-helix formed by residues 5–17 (see Fig. 5b). The Ala residues at positions 7 and 13 are located inside the α-helical region, which may stabilize the helical structure of G2,7,13A.

SMAP-18 forms a small hydrophobic core consisting of Leu^3^, Leu^6^, Ile^10^, and Val^14^, with positively charged residues surrounding the hydrophobic core (see Fig. 5a). In contrast, G2,7,13A forms an almost perfect amphipathic structure as the positively charged residues gather on one side of the helix and the hydrophobic residues gather on the other side (see Fig. 5b). Structural studies suggest that the conformational transition occurs through the substitution of Gly residues with Ala and this structural difference affects the antimicrobial activity and/or mechanism of action of the peptides. We therefore compared this activity and mode of action using various mechanism experiments.

### Cytoplasmic membrane electrical potential

**Figure 6:**
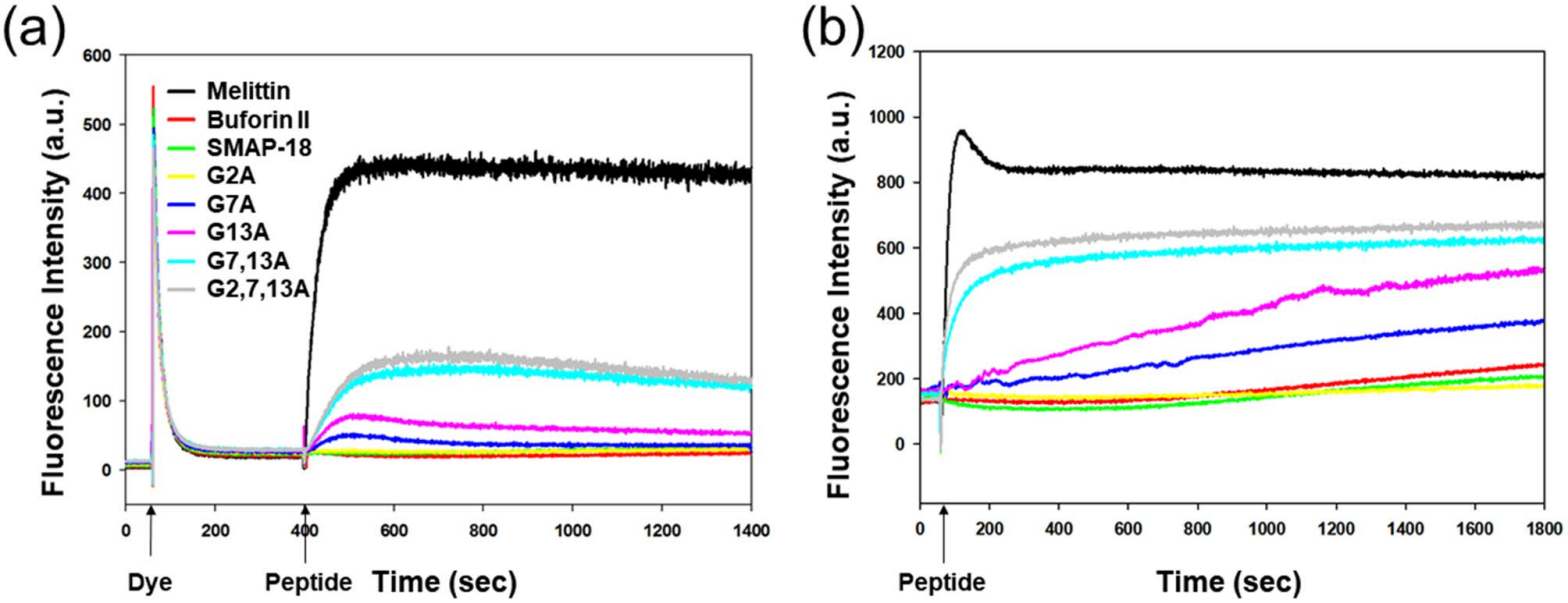
Mechanism assay of SMAP-18 peptides. (**a**) Time-dependent membrane depolarization of *S. aureus* at 2 × MIC. (**b**) SYTOX Green uptake assay induced by the SMAP-18 peptides at 2 × MIC.

To measure the ability of the peptides to depolarize *S. aureus* membranes, diSC_3_-5 dye was used. Under the influence of a membrane potential, the dye can accumulate in the cytoplasmic membrane and cause a self-quenching of fluorescence. When the membrane potential is destroyed, the dye is dissociated into the buffer to increase the fluorescence intensity. Figure 6a shows that membrane depolarization was monitored over a period of 1400 s for SMAP-18 and its analogs at 2 × MIC. Similar to buforin II (an intracellular-targeting AMP) when used as a negative control AMP, SMAP-18 and G2A did not induce membrane depolarization. In contrast to even a weak membrane-targeting AMP like melittin as the positive control, G7,13A and G2,7,13A induced a significant membrane depolarization. G7A and G13A induced a minimal increase in fluorescence.

### SYTOX Green uptake assay

SYTOX Green, a DNA-binding dye, becomes fluorescent when bound to nucleic acids and only enters cells with a compromised plasma membrane. As shown in Fig. 6b, SMAP-18 and G2A did not increase the fluorescence intensity at 2 × MIC like the activity of buforin II. In contrast, similar to melittin, G7,13A and G2,7,13A induced the maximal fluorescence upon treatment of the peptide, thus indicating rapid membrane disruption. G7A and G13A induced a little increased fluorescence, similar to that seen through membrane depolarization.

### Confocal laser scanning microscopy

**Figure 7:**
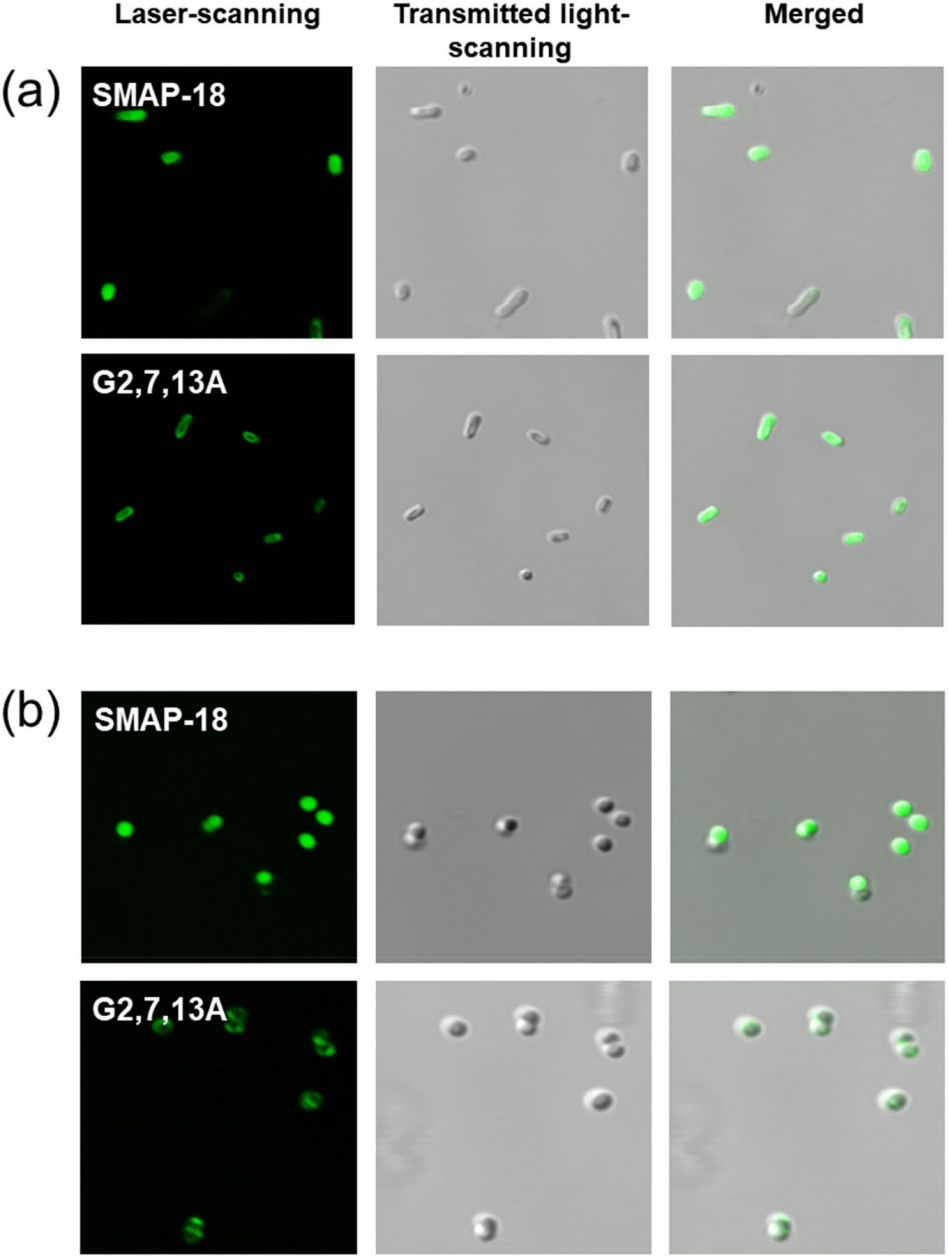
Confocal laser scanning microscopy of *E. coli* (**a**) and *S. aureus* (**b**) treated with FITC-labeled peptides.

To find out where the peptides act within the bacteria, fluorescein isothiocyanate (FITC)-labeled SMAP-18 and G2,7,13A were incubated with gram-negative *E. coli* and gram-positive *S. aureus*; their localization was visualized by using confocal laser scanning microscopy (see Fig. 7). SMAP-18 completely penetrated the bacterial cell membrane and accumulated in the cytoplasm of *E. coli* (see Fig. 7a) and *S. aureus* (see Fig. 7b). In contrast, G2,7,13A likely bound to the cell membrane since the fluorescence intensity is much weaker at inside of the cell compared to SMAP-18 in both *E. coli* and *S. aureus*.

## Discussion

The structural features of AMPs are probably correlated with their biological activities and/or the mode of their action. The antimicrobial activity of helical AMPs is thought to be determined by global structural parameters rather than by specific amino acid sequences^28^. In this study, we showed that the structural transition of SMAP-18 by Gly→Ala substitution altered its mode of action. The Ala mutations at Gly residues (i.e., G2A, G7A, G13A, G7,13A, and G2,7,13A) of SMAP-18 did not significantly alter their antimicrobial activities; however, the action mechanism of SMAP-18 was changed by Ala substitutions at Gly^7^ and Gly^13^. In particular, G2,7,13A increased the antimicrobial activity for most bacteria, but slightly reduced for *S. epidermidis*. These results indicated that the antimicrobial activity of G2,7,13A depends on the types of bacterial strains and can provide strain-specific information for the design of novel peptide-based antibiotics. The short forms by N- or C-terminal truncation greatly reduced their antimicrobial activity. In addition, the truncated analogs did not form helical structure even in a membrane-mimicking environment, suggesting the entire sequence of SMAP-18 is critical for both antimicrobial activity and formation of the helical structure.

The mechanism of SMAP-18 in this study, based on fluorescence experiments of SMAP-18 and its analogs, revealed that the analogs interact with bacterial membrane with higher binding affinity than the wild-type SMAP-18, then the analogs effectively depolarize/destabilize *S. aureus* membrane and accumulate on *E. coli* membrane. In addition, calcein dye leakage and ONPG assays also suggested that the wild-type SMAP-18 seem to have a similar mechanism of action to buforin II (an intracellular targeting AMP), while the analogs such as G2,7,13A acts more like melittin (a membrane-targeting AMP) (supplementary Fig. S3).

A comparison of the tertiary structure of SMAP-29 and SMAP-18 reveals that the N-terminal region from Arg^1^ to Gly^18^ of SMAP-29 is very flexible, in particular the hinge region is present in Gly^7^ and the region from Arg^8^ to Tyr^17^ is near helical structure. In contrast, SMAP-18 takes an entirely bent shape and contains the amphiphilic α-helical structure with narrow regions from Gly^7^ to Ile^10^.

A previous study has reported that SMAP-29 has two binding sites for LPS at the N-terminus and C-terminus, with the N-terminal binding site having a higher affinity for LPS than the C-terminal binding site. The presence of multiple binding sites allows SMAP-29 to bind LPS with high affinity; however, SMAP-18 did not induce any inhibitory activity of tumor necrosis factor-α (TNF-α) production from LPS-stimulated RAW 264.7 cells and did not damage the membrane^24^. This may be due to structural differences between the N-terminals (i.e., residues 1–18) of SMAP-29 and SMAP-18. Interestingly, the amphiphilic α-helices and bent structure of SMAP-18 are similar to previously reported buforin II structures^25^ and play an important role in the mechanism by which these peptides enter the bacterial cells. However, the N-terminal part of SMAP-29 is not amphipathic and has a hinge region at Gly^7^. It is therefore expected to have other functions, such as LPS binding, rather than the intracellular mechanism of entry into bacterial cells. SMAP-29 has been known to be very cytotoxic to mammalian cells. We therefore compared the hemolytic activity of SMAP-29, SMAP-18, and G2,7,13A (Supplementary Fig. S4) showed about 60% hemolysis activity at 100 μM concentration, but SMAP-18 and G2,7,13A showed very low hemolytic activity about 12 and 2%, respectively.

**Figure 8:**
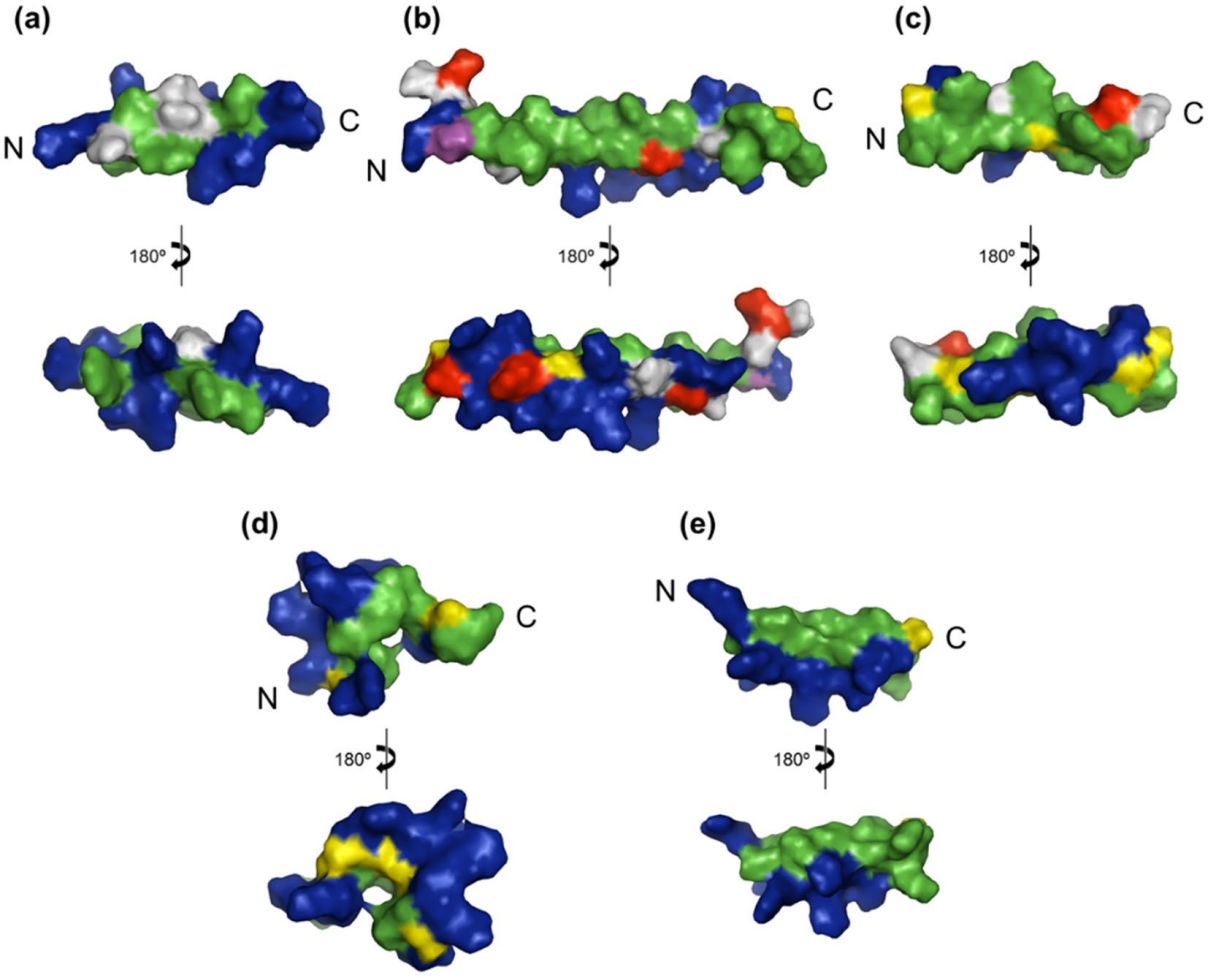
Surface structural characteristics of penetratin (**a**), LL-37 (**b**), magainin 2 (**c**), SMAP-18 (**d**), and G2,7,13A (**e**). The amino acids residues are colored as follow: positively charged residues in blue; negatively charged residues in red; hydrophobic residue in green; glycine in yellow.

As shown in Fig. 8, penetratin, a cell penetrating peptide, has a hydrophobic surface of 480.3 Å^2^ and is partially present in the N-terminal part of the peptide (see Fig. 8a). In contrast, the hydrophobic surfaces of LL-37 and magainin 2, known as membrane-disrupting peptides, are 1439.8 Å^2^ and 1129.7 Å^2^, respectively; these are widely present on one side of the α-helical axis (see Fig. 8b,c). The intensity of the membrane damage of the peptides apparently depends on the area of hydrophobic surface of the peptide. The area of hydrophobic surface of SMAP-18 is 607.9 Å^2^ and hidden by the bent structure (see Fig. 8d). SMAP-18 thus cannot strongly bind to the hydrophobic region of the membrane and can enter into the cell without damaging the membrane. G2,7,13A, in contrast, has stronger hydrophobic interactions with the lipid hydrophobic tail of the membrane because the hydrophobic surface of G2,7,13A is 789.1 Å^2^, wider and is totally exposed to the membrane surface compared to wild-type SMAP-18 (see Fig. 8e).

CD analysis revealed that the helicity of G13A mutant increased significantly, suggesting that Gly^13^ seems to break the helical structure of SMAP-18 peptide. Gly^13^ plays an important structural role in forming the bent structure of SMAP-18. Since Gly^13^ is located in the bent part of the structure of SMAP-18, CD spectra show that the helicity of peptide was significantly increased by the substitution for Gly^13^ by Ala. In addition, the substitution of Gly^7^ with Ala results in an amphipathic α-helix structure; these peptides show a mechanism different from wild-type SMAP-18. This result suggests that the short length of the α-helix and the bent structure of SMAP-18 is important for it to penetrate the bacterial cell membrane.

**Figure 9:**
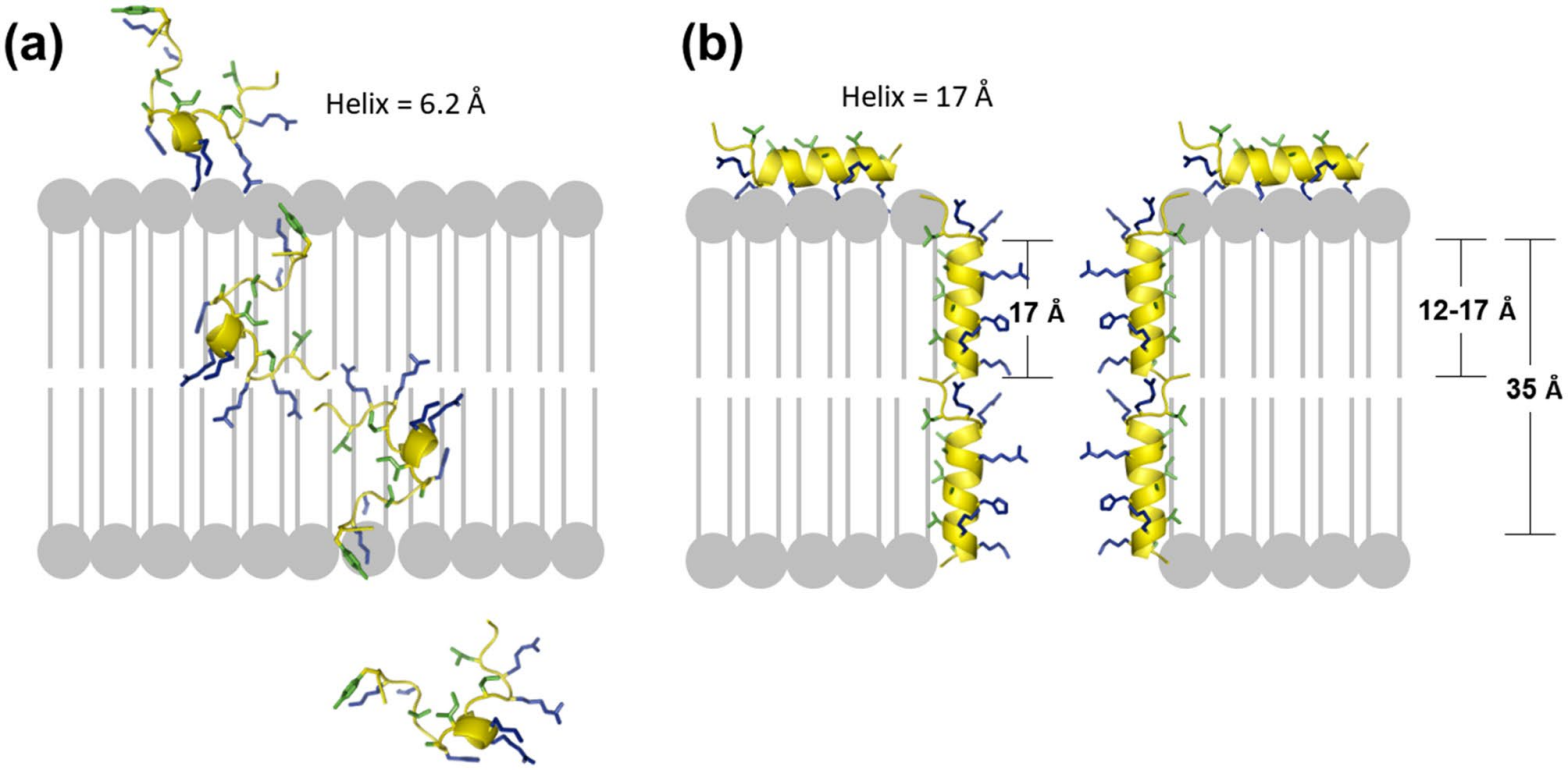
Schematic representation of the action of SMAP-18 (**a**) and G2,7,13A (**b**) after membrane interaction. SMAP-18 penetrates the cellular membrane without any disruption. G2,7,13A binds to the membrane lipid bilayer and change the integrity of the membrane structure.

Analysis of the interaction between peptides and the cell membrane found the following: the cation region of SMAP-18 or G2,7,13A adsorbs to the cell membrane and the hydrophobic residues of the α-helices are separated along the axis of the α-helix to act effectively with the cell membrane. The α-helix length of SMAP-18 is only 6.2 Å, which is not long enough to interact with the lipid tail region (see Fig. 9). In contrast, the α-helix length of G2,7,13A is 17.0 Å, similar to the length of the tail portion of the phospholipid. This is why the amphipathic α-helix length of G2,7,13A is believed to be sufficient to interact with the bacterial membrane.

## Conclusions

In this study, we determined the NMR structure of SMAP-18 peptides and identified the structural and functional residues that play important roles in the conformational change and mechanism of action of SMAP-18. We focused on Gly residues of SMAP-18; five analogs were synthesized through Gly → Ala substitution (i.e., G2A, G7A, G13A, G7,13A, and G2,7,13A), which altered the helical content of the peptides. We found that G7,13A and G2,7,13A changed their mode of action and molecular conformation from random to an α-helical structure. These results suggested that Gly residues especially at positions 7 and 13 in SMAP-18 may be involved in the structural and functional changes of SMAP-18. In order to understand more accurate structure and activity relationship, it seems necessary to study the structure of SMAP-18 peptides in an environment more similar to the bacterial membrane.

## Experimental

### Peptide synthesis

Peptides were synthesized by solid-phase 9-fluorenylmethoxycarbonyl (Fmoc) chemistry on rink amide 4-methylbenzhydrylamin (MBHA) resin, using protected amino acid and N,N’-diisopropylcarbodiimine (DIC) and hydroxybenzotriazole (HOBt) as coupling agents. FITC-labeled peptides were additionally synthesized with Fmoc-ε-Ahx-OH in the N-terminal region. After synthesis, FITC was added to the synthesized peptides with diisopropylethylamine (DIEA) and samples were shaken overnight at room temperature. The synthesized peptides were cleaved from the resin with trifluoroactic acid (TFA)/H_2_O/ethanedithiol/phenol and thioansole (percentage volume ratios: 82.5/5/2.5/5/5) for 2 h at 25 °C, after which the crude peptide was washed and precipitated using 10 times the volume of cold diethyl ether. Crude peptides were purified through preparative reverse-phase high-performance liquid chromatography (RP-HPLC) (LC-6AD, Shimadzu, JAPAN) with a Shim-pack C18 column (dimensions: 20 mm × 250 mm). The mobile phase components were 0.05% TFA in water (solvent A) and 0.05% TFA in acetonitrile (ACN) (solvent B). The gradient for the purification of peptides was 5 to 35% of solvent B for 30 min. The final purity of peptides was confirmed using analytical RP-HPLC and triple-quadrupole equipped electrospray ionization (ESI)-LC–MS (API2000, AB SCIEX, US). All compounds are > 95% pure by HPLC analysis.

### CD spectroscopy

CD spectra were measured on a J-810 spectropolarimeter (JASCO) using a 1.0 mm path-length cell. The SMAP-18 and SMAP-18 analogs were dissolved in distilled H_2_O (10 mM sodium phosphate, pH 7.0) and 50% TFE; the peptide concentration was 100 μM. Three scans were conducted from 190 to 260 nm and the data are expressed in terms of mean residue molar ellipticity [θ]_mr._ The mean residue ellipticity, [θ]_mr_ (deg cm^2^ dmol^-1^), was calculated according to the equation [θ]_mr_ = (100θ)/(clN), where θ is the measured ellipticity (mdeg), c is the sample concentration (mM), l is the path length (cm), and N is the number of amino acids. Percent α-helicity was calculated using the equation ([θ]_mr222nm_—3000)/(− 39,000 × 100)^29^. Reference values for [θ]_mr222nm_ = 0 and 36,000 (deg cm^2^ dmol^−1^) were used for 0% and 100% helicity, respectively^30^.

### Determination of minimal inhibitory concentration (MIC)

The antimicrobial activity of the peptides, i.e., their MIC values, were determined against *Escherichia coli* (KCTC 1682), *Salmonella typhimurium* (KCTC 1926), *Pseudomonas aeruginosa* (KCTC 1637), *Bacillus subtilis* (KCTC 3068), *Staphylococcus epidermidis* (KCTC 1917), *Staphylococcus aureus* (KCTC 1621), and Methicillin-resistant *Staphylococcus aureus* (CCARM 3089, 3090 and 3095) using broth microdilution in a 96-well plate according to existing methodologies^23^. Bacteria were grown at 37 ℃ on a spinner culture until a mid-log phase diluted to 2 × 10^6^ CFU/ml in 1% peptone. The bacterial suspension was added to 100 μl of the sample solution (i.e., serial twofold dilution in 1% peptone). After incubation for 16 h at 37 ℃, the MIC value was determined as the lowest concentration of sample solution in cells with no bacterial growth. All experiments were performed in triplicate and included growth and sterility controls.

### Membrane depolarization assay

The cytoplasmic membrane depolarization activity of the peptides was measured using the membrane potential sensitive dye diSC_3_-5, as previously described^31,32^. Briefly, *S. aureus* (KCTC 1621) was grown at 37 ℃ with agitation until the mid-log phase (OD_600_ = 0.3) and was harvested by centrifugation. Cells are washed twice with a buffer solution (i.e., 20 mM Glucose, 5 mM HEPES, 100 mM KCl; pH 7.4) and were resuspended to an OD_600_ = 0.05 in the buffer. Membrane depolarization was monitored by recording changes in the intensity of fluorescence emission of diSC_3_-5 after peptide concentration. Leakage experiments were performed on a F-4500 FL spectrophotometer (Hitachi, Japan) with excitation and emission wavelengths of 622 nm and 670 nm, respectively.

### SYTOX Green uptake assay

The membrane permeabilization activity of the peptides was determined by using the SYTOX Green uptake assay, as described previously^33^. *S. aureus* (KCTC 1621) grown at 37 ℃ until the mid-log phase (OD_600_ = 0.4). The cell pellet was washed using PBS (pH 7.4) and was diluted to an OD_600_ = 0.05 in PBS (pH 7.4); SYTOX was added to the final concentration of 0.1 μM and this solution was pre-incubated in the dark at 4 ℃ for 16 h. Fluorescence was monitored using a F-4500 FL spectrophotometer (Hitachi, Japan) and excitation and emission wavelengths of 480 nm and 520 nm, respectively.

### Confocal laser microscope

*E. coli* (KCTC 1682) and *S. aureus* (KCTC 1621) were grown at mid-log phase and washed three times with PBS (pH 7.4). The bacteria were diluted at 10^6^ CFU/ml and incubated with FITC-labeled peptides (10 μM) at 37 ℃ for 30 min. After incubation, this solution was washed thrice with PBS and was then transferred to a confocal dish. The FITC-labeled was peptides were observed using TCS SP5 AOBS Tandem (Leica, Germany). Fluorescent images were obtained with a 488 nm bandpass filter for FITC excitation.

### NMR spectroscopy

NMR measurements were performed using a Bruker AVANCE 600 spectrometer equipped with an XYZ gradient triple-resonance probe. The samples for NMR experiments were 2 mM for SMAP-18 and G2,7,13A, dissolved in 10 mM sodium phosphate buffer (pH 6.0) and 50 mM NaCl containing 50% TFE-d3 and 10% D_2_O at 298 K. TOCSY spectra were recorded using a MLEV-17 pulse scheme with isotropic mixing times of 70 ms. NOESY spectra were obtained with a mixing time of 300 ms. Suppression of the solvent resonance in both the NOESY and TOCSY spectra was achieved using the WATERGATE scheme^34^. DOQ-COSY spectra were collected with water pre-saturation and NMR data were processed using NMRPipe^35^ and NMRView^36^.

### Structural estimation

NOE resonance assignments and initial NOE constraints were obtained from CYANA 2.1 using CANDID^37^. To expedite the automated assignment of additional NOE restraints by CANDID, hydrogen bonds were added to the α-helical region of the peptides. Structural calculations of the peptides were carried out by following the standard protocol of CYANA calculations. This study arrived at 100 final structures, of which 20 structures with the lowest target function value were selected. All images were prepared by using MOLMOL^38^ and Pymol programs.

### Statistical analysis

All statistical analysis in this study was performed with SPSS 16.0 software using one-way analysis of variance (ANOVA). The data are expressed as the means ± standard deviation (SD) from at least three independent experiments. Differences with a P-value of less than 0.05 were considered statistically significant.

## Supplementary Information

Supplementary Information.

## Data Availability

The datasets used and/or analysed during the current study available from the corresponding author on reasonable request.
