# Supplementary material for: Discovery of structural and functional transition sites for membrane-penetrating activity of sheep myeloid antimicrobial peptide-18
Source: Sci Rep. 2023 Jan 23;13:1238. doi: 10.1038/s41598-023-28386-6 (PMC9871035; doi:10.1038/s41598-023-28386-6)
Supplement: Supplementary file 1 — Supplementary Information. [file 41598_2023_28386_MOESM1_ESM.docx]

**Supplementary Material**

**Discovery of Structural and Functional Transition Sites for Membrane-Penetrating Activity of Sheep Myeloid Antimicrobial Peptide-18**

Bomi Jung^1^, Hyosuk Yun^1^, Hye Jung Min^2^, Sungtae Yang^3^, Song Yub Shin^4^*, Chul Won Lee^1^*

*^1^Department of Chemistry, Chonnam National University, Gwangju, 61186, Republic of Korea*

*^2^Department of Cosmetic Science, Gwangju Women's University, Gwangju, 62396, Republic of Korea*

*^3^Department of Microbiology, School of Medicine, Chosun University, Gwangju, 61452, Republic of Korea*

*^4^Department of Cellular & Molecular Medicine, School of Medicine, Chosun University, Gwangju, 61452, Republic of Korea*


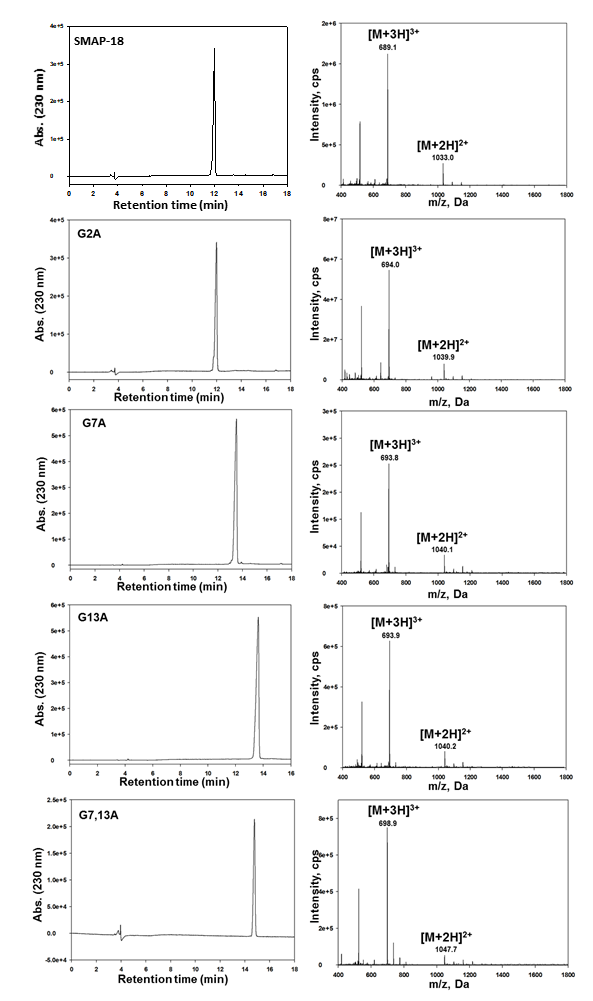
Figure S1 (*continued*)


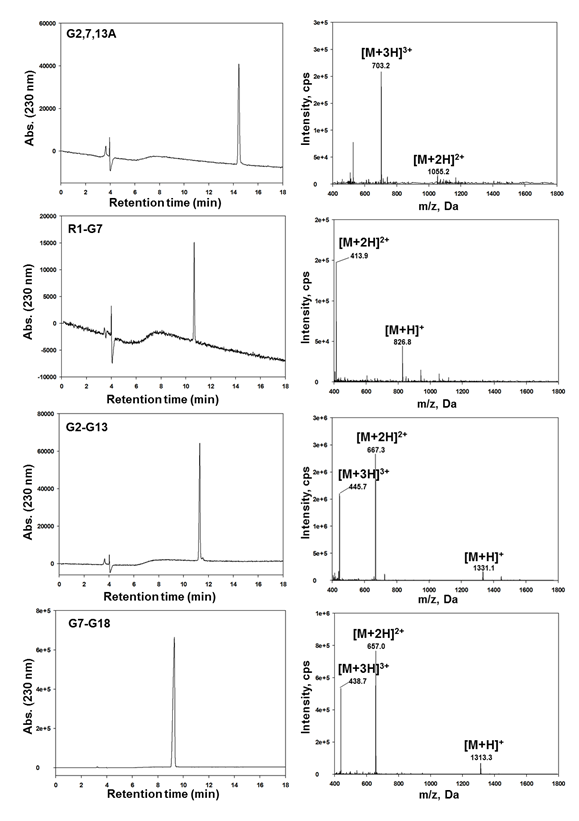


**Figure S1.** LC-MS analysis of the purified SMAP-18 and its analogs: HPLC analysis (*left*), ESI-MS (*right*)


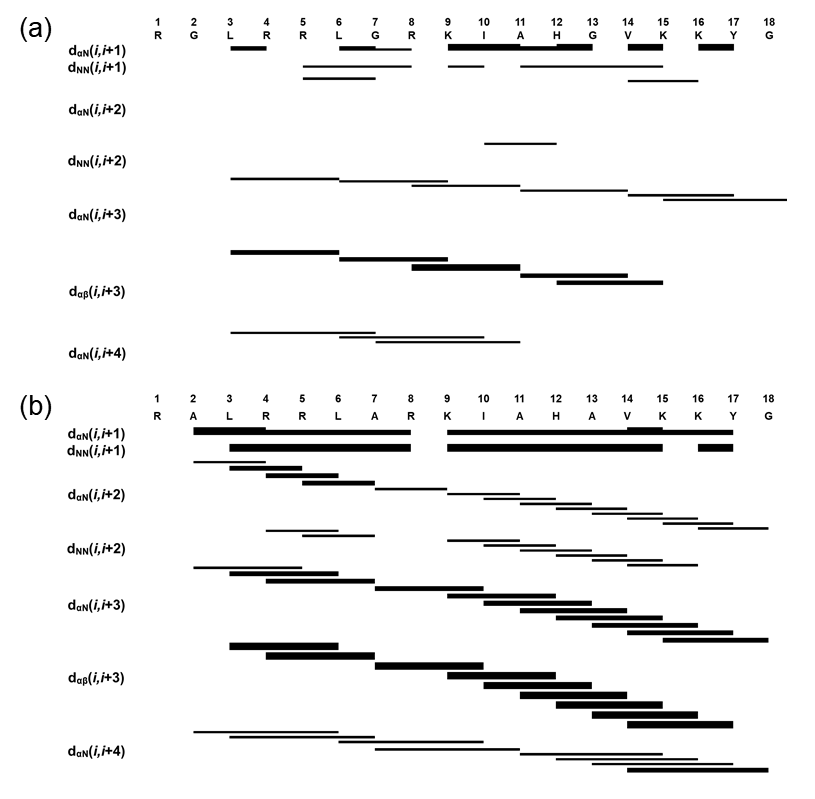


**Figure S2.** Sequential and medium-range NOEs of SMAP-18 (a) and G2,7,13A (b) observed in the NOESY spectra recorded in the presence of 50 % TFE. The thickness of the bars is related to the NOE cross-peak intensities (weak, medium and strong).


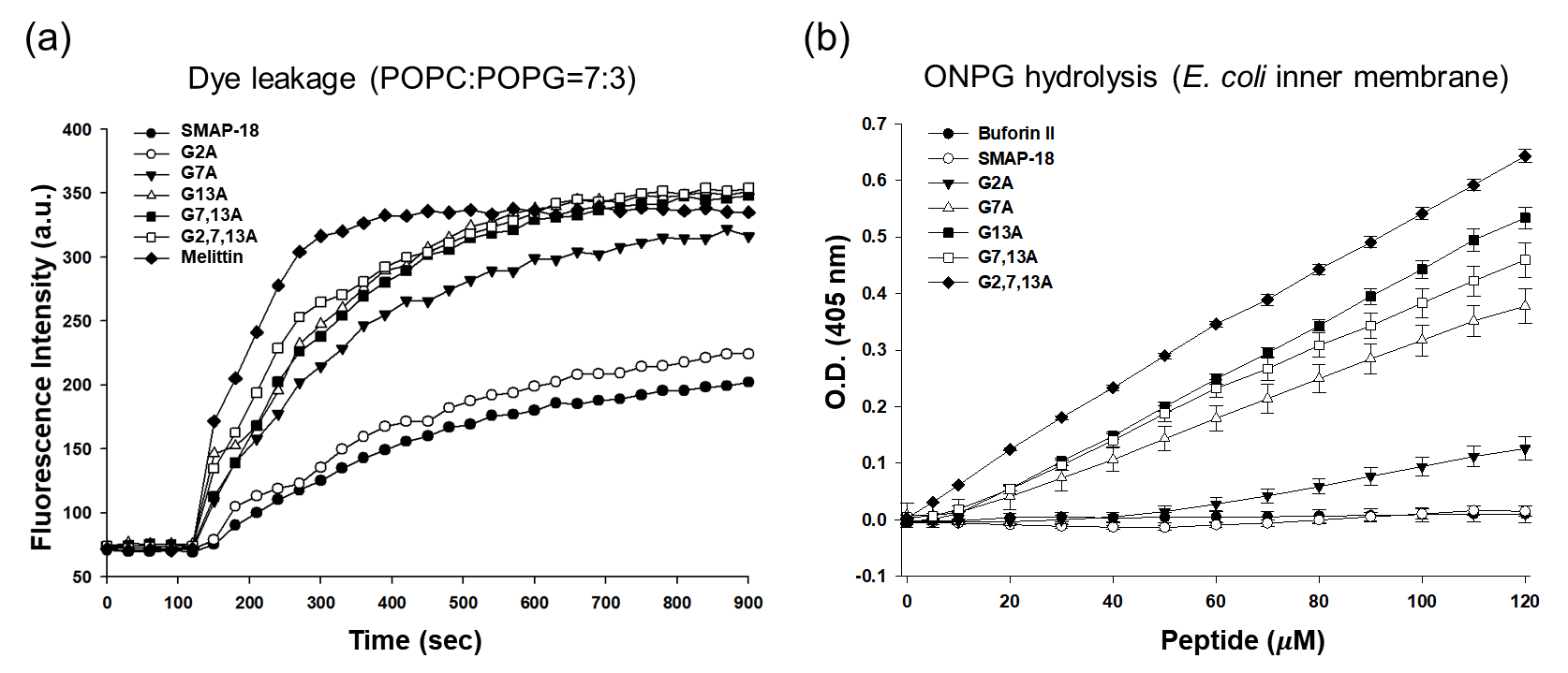


**Figure S3**. Calcein dye leakage (a) and ONPG (b) assays. To determine the ability of peptides to permeabilize the bacterial membrane models, large unilamellar vesicles (LUVs) composed of egg yolk phosphatidylethanolamine (EYPE)/egg yolk phospha- tidylglycerol (EYPG) (7:3, w/w) entrapped with calcein dye. Peptide-induced membrane permeability was measured (excitation λ = 490 nm, emission λ = 520 nm) by increase in ﬂuorescence intensity of calcein released from LUVs upon addition of 2×MIC of peptides. Membrane-active peptides will instantly perturb the LUVs and cause a rapid release of the dye. Complete calcein release was obtained by using 0.1% Triton X-100. Inner membrane permeability of peptides was assessed by measuring the release of *β*-galactosidase from *E. coli* ML-35 using ONPG (*o*-nitrophenyl-*β*-galactosidase) a nonchromogenic substrate for cytoplasmic *β*-galactosidase enzyme. Brieﬂy, mid-log phase of *E. coli* ML-35 were suspended to an A600nm of 0.5 in sample buffer (10 mM sodium phosphate, 100 mM NaCl, pH 7.4) containing 1.5 mM ONPG. The permeabilization of the inner membrane after addition peptides was assessed spectrophotometrically at 405 nm. Increase in ﬂuorescence indicates the hydrolysis of ONPG to *o*-nitrophenol. The inner membrane permeability was determined by the inﬂux of ONPG, that was subsequently cleaved into the yellow product *o*-nitrophenol by *β*-galactosidase in the cytoplasm.

**Figure S4**. Hemolysis of sheep red blood cell by SMAP-29, SMAP-18, and G2,7,13A peptides (0 − 100 μM).
